# Synergistic Effect and Enhancement Mechanism of Foam Concrete Composites by Incorporating Aerogel, Hollow Glass Microspheres and Nano-Silica

**DOI:** 10.3390/ma19050990

**Published:** 2026-03-04

**Authors:** Kaihe Dong, Sili Chen, Junxiang Wang, Xinxin Shi, Jingyu Zhang, Jinzhu Meng

**Affiliations:** 1 School of Materials Science and Engineering, Shenyang University of Technology, Shenyang 110870, China; dongkaihe1988@126.com; 2 School of Applied Technology, Anshan Normal University, Anshan 114056, China; 3 School of Architecture and Civil Engineering, Shenyang University of Technology, Shenyang 110870, China; wangjunxiang@sut.edu.cn (J.W.); zjy7019@sut.edu.cn (J.Z.); jinzhumeng@sut.edu.cn (J.M.); 4 School of Civil Engineering, University of Science and Technology Liaoning, Anshan 114051, China; shixxin94@126.com

**Keywords:** nanomaterials, mechanical properties, effective porosity, mechanism analysis

## Abstract

Aerogel-incorporated foam concrete has attracted significant attention in the construction sector owing to its light weight and superior thermal insulation properties. Nevertheless, its practical application in external wall insulation systems is hindered by the high cost of aerogel (AG) and the inherent trade-off between thermal efficiency and mechanical strength. To overcome these limitations, this study introduces a composite design that partially replaces AG with low-cost hollow glass microspheres (HGMs) and incorporates nano-silica (NS) as a strengthening agent. Foam concrete specimens with a constant dry density of 700 kg/m^3^ were fabricated with these additives. Through an orthogonal experimental approach, the synergistic effects of AG, HGMs, and NS on mechanical properties, porosity, water absorption, and durability were systematically evaluated. The results demonstrated that 4% AG content significantly reduced effective porosity by 33% and water absorption by 59%, while 4% HGM increased compressive and flexural strength by 13.5% and 19.7%, respectively. The addition of 2% NS further enhanced mechanical performance, yielding 25.9% and 21.6% improvements in compressive and flexural strength. The optimal formulation (A4H4N2) effectively balanced thermal insulation and mechanical properties, offering a viable strategy for producing cost-effective, high-performance foam concrete suitable for building envelope applications.

## 1. Introduction

The severe threat of global climate change has increased the universal importance of energy conservation and emission reduction where carbon neutrality is an urgent priority [1,2]. The construction industry, as a major global energy-consuming sector, is an essential source of potential energy savings. Reducing heat loss through building envelopes represents a key research direction in the development of energy-efficient buildings [3,4].

AG foam concrete, a novel building material characterized by a lightweight and porous structure, is an important component in building energy efficiency, road engineering, and a range of other applications due to its superior thermal insulation, sound absorption, and seismic resistance properties. However, several technical challenges limit its practical application. First, the high cost of AG substantially increases material expenses while compromising the overall mechanical strength of the composite. Second, both AG and foam concrete inherently exhibit low mechanical strength, making it difficult to satisfy load-bearing requirements while maintaining high thermal insulation performance [5,6]. Third, foam concrete’s high porosity leads to low mechanical strength, poor heat resistance, high water absorption, significant shrinkage, and inferior durability [7]. These drawbacks severely restrict the broad application of AG foam concrete in building envelopes.

To address these challenges, two complementary strategies are essential: identifying cost-effective alternatives to AG for thermal insulation and incorporating materials that enhance the mechanical properties of foam concrete.

Regarding cost-effective alternatives, HGMs have emerged as a promising candidate. Also referred to as glass lightweight microspheres, HGMs are microscale hollow particles characterized by low density and high strength [8,9]. Composed primarily of amorphous SiO_2_ and made from soda-lime borosilicate glass, HGMs offer advantages including low density, high crush strength, and pozzolanic reactivity. These properties make them widely studied for producing lightweight, high-strength cementitious materials [10,11]. Kaihe Dong et al. [12] demonstrated that HGMs significantly enhance the thermal insulation performance of foam concrete composites (FCC). This improvement is particularly notable in moisture-containing foam concrete. In a separate study, Wen Yang et al. [13] reported that the synergistic effect of HGMs and AG can further increase the thermal insulation properties of cement-based materials. Therefore, partially substituting AG with HGMs may represent a viable strategy to improve the economic feasibility of AG in foam concrete applications.

In terms of enhancing mechanical properties, NS has demonstrated significant potential. As an emerging nanoscale material with particle sizes below 20 nm, NS exhibits multiple beneficial effects, including nucleation effects, pozzolanic activity, and morphological effects, all of which contribute to improved concrete performance [14,15,16]. Well-dispersed nanoparticles create a favorable environment for cement hydration and crystallization, accelerating the hydration process [17]. Uniformly distributed nanoparticles promote the formation of smaller crystals, resulting in a denser microstructure that reduces crack initiation and propagation [18]. Consequently, the hardened material exhibits enhanced mechanical properties. Studies have confirmed that incorporating NS can improve both the mechanical performance and durability of concrete [19].

Despite the demonstrated potential of AG, HGMs, and NS in enhancing individual aspects of foam concrete performance, systematic investigations into their combined effects remain limited. In particular, the synergistic enhancement mechanisms among these three components—especially regarding waterproof properties (e.g., volumetric water absorption and softening coefficient) and pore structure characteristics—have not been adequately addressed in the literature.

To fill this research gap, the present study employed an orthogonal experimental design to systematically investigate the influence of varying dosages of AG, HGM, and NS on the mechanical properties, pore structure, volumetric water absorption, and softening coefficient of FCC. A nonlinear mathematical model was established to characterize the relationship between effective porosity and compressive/flexural strength. Furthermore, microstructural evolution and hydration product characteristics were analyzed using scanning electron microscopy (SEM) and X-ray diffraction (XRD). The optimal mix proportion was identified, and the synergistic interaction and enhancement mechanisms of the AG-HGM-NS composite system were elucidated. This research aims to provide theoretical support and practical guidance for the material design and engineering application of high-performance FCC.

The novelty of this study lies in its systematic investigation of the ternary AG-HGM-NS system, which advances beyond existing studies that examine these materials individually or in partial combinations. While previous research has focused on the independent effects of these components on specific properties, our work reveals their synergistic interactions and identifies an optimal balance among thermal insulation, mechanical strength, and waterproof performance—a holistic optimization that has not been previously achieved. By elucidating the synergistic mechanisms among these three components, this work contributes to the development of cost-effective, high-performance foam concrete materials suitable for building envelope applications.

## 2. Raw Materials and Experiment Procedures

### 2.1. Materials

**Figure 1:**
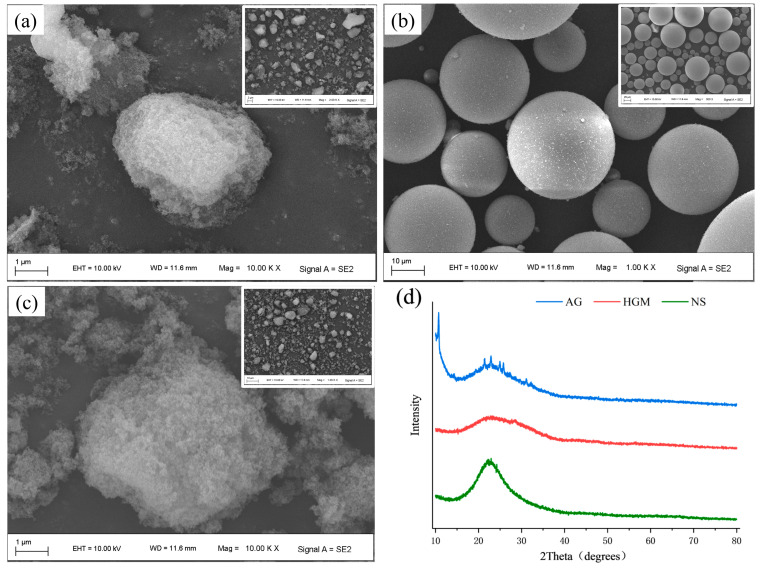
Scanning electron microscopy images of (**a**) AG, (**b**) HGM and (**c**) NS and (**d**) XRD patterns of AG, HGM and NS.

This study employed hydrophobic materials including nanoporous SiO_2_ AG (<5 μm particle size, <15 nm pores, >90% porosity, 400–700 m^2^/g surface area), hydrophobic NS (as received from Zhengda Nano Technology Co., Ltd. (Ningbo, China), surface hydrophobized with KH550, 30 nm average size, 200 ± 50 m^2^/g surface area), and hydrophobic HGM (55–95 μm particle size, 2.07 MPa compressive strength). The SEM and XRD characterization results are shown in Figure 1. A 42.5-grade sulfoaluminate cement complying with the Chinese national standard GB/T 20472-2006 Sulphoaluminate cement [20] was used in the experiments (Table 1). Physical foaming was carried out using hydroxypropyl methylcellulose (HPMC) as both a foam stabilizer and a viscosity modifier (Table 2). The foaming agent employed was YS-10 cement foaming agent, manufactured by Beijing Yashe Building Materials Technology Co., Ltd. (Beijing, China), which was diluted with deionized water at a ratio of 1:30. Foam was generated using a JZ-01 foaming machine (Excellent Machinery and Equipment Mall, Xingtai, China) via the physical foaming method. The foaming pressure of the machine was approximately 1.0 MPa, and the foam could be transported horizontally up to 15–30 m and vertically up to 3–6 m. The resulting foam density was 45 kg/m^3^.

### 2.2. Fabrication of FCC

The experiment aimed to prepare foamed concrete with a target density of 700 kg/m^3^. Three factors—AG, HGM, and NS—were selected, each set at five levels: 0%, 1%, 2%, 3%, and 4% (by mass of cement). A three-factor, five-level L_25_(5^3^) orthogonal array was adopted for the design, with a fixed water-to-binder ratio of 0.8, resulting in a total of 25 test groups. The orthogonal experimental layout and corresponding results are presented in Table 3. For each mixture proportion, the reported values are the mean results, with sample sizes of *n* = 3 for compressive strength and *n* = 6 for flexural strength. Variance analysis was performed on the experimental results, and the outcomes are summarized in Table 4.

**Figure 2:**
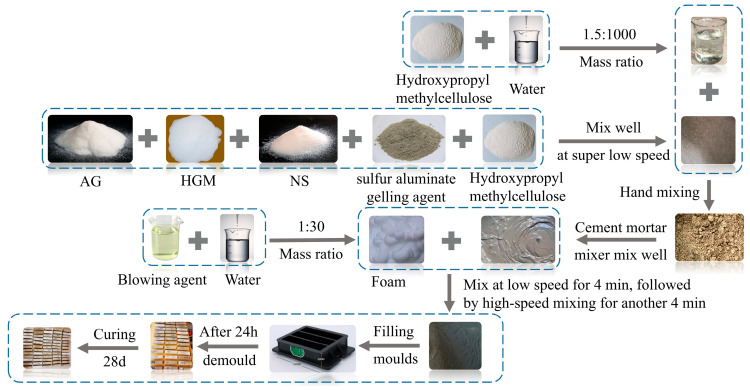
Flowchart of the preparation procedure.

The sample preparation procedure, illustrated in Figure 2, required careful sequencing due to the use of nano- and micron-sized hydrophobic materials. First, a thickening solution was prepared by mixing HPMC with water at a mass ratio of 1.5:1000 at room temperature, followed by standing for 15 min. Dry materials—cement, AG, HGM, NS, and foam stabilizer—were manually blended into a uniform mixture. After adding the thickening solution, mixing was initiated at an ultra-low speed, after which a mechanical mixer was used with gradually increasing speed, then used a mechanical mixer with gradually increased speed to achieve homogeneity. Concurrently, foam was generated using a foam generator and gradually incorporated into the slurry. Initial low-speed mixing ensured even foam distribution, followed by high-speed mixing to homogenize the final mixture.

### 2.3. Test Methods

#### 2.3.1. Mechanical Strength Test

Mechanical performance tests were conducted using a WDW-50 electronic universal testing machine (Wuxi Jianyi Instrument Machinery Co., Ltd., Wuxi, China) with a maximum load capacity of 50 kN and a loading accuracy of ±0.5%. Specimens were prepared in strict accordance with ISO 679:2009 [21]. For each test group, three prismatic specimens with dimensions of 40 mm × 40 mm × 160 mm were molded. The specimens were demolded after 24 h and cured under standard conditions (20 ± 2 °C, relative humidity ≥ 95%) until the designated testing ages of 3, 7, and 28 days.

**Figure 3:**
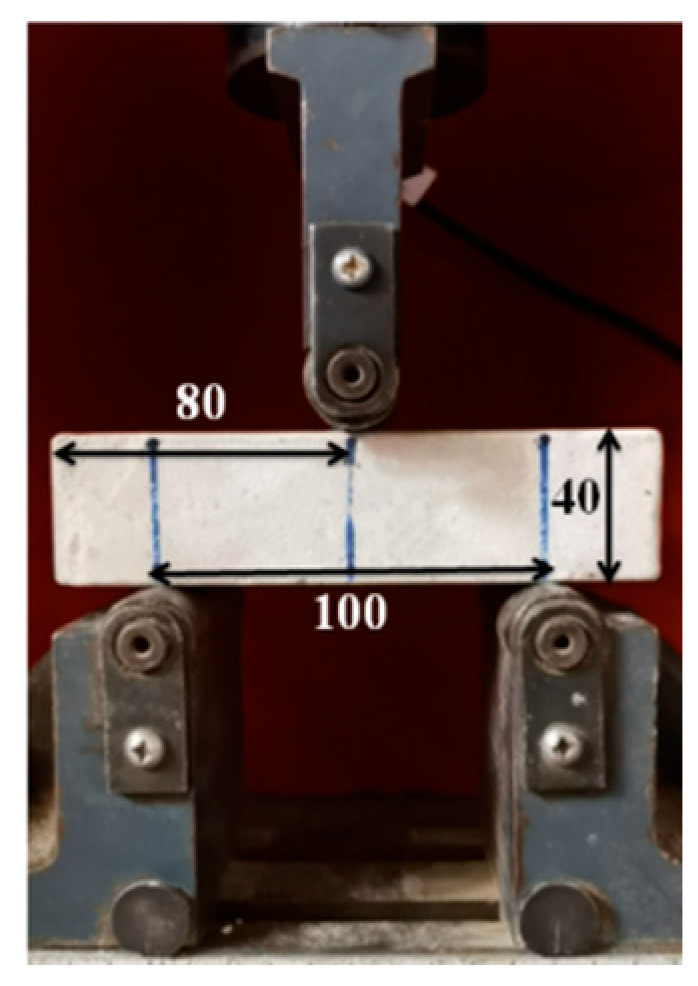
Schematic representation of the three-point bending test.

**Figure 4:**
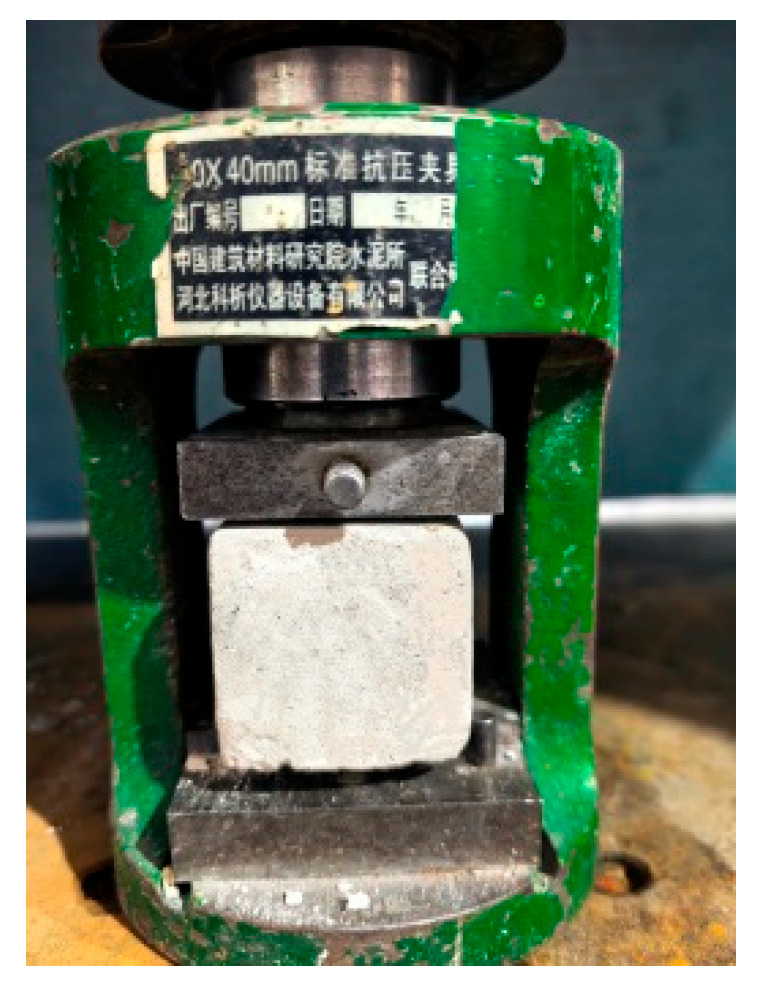
Compressive strength test.

The flexural strength test was conducted using ISO 679:2009 [21] using the three-point bending method with a span of 100 mm, as illustrated in Figure 3. The flexural strength was calculated according to Equation (1), and the result was taken as the arithmetic mean of the measurements from the three specimens. The compressive strength test was performed in accordance with ISO 679:2009 [21] using the six half-specimens obtained from the flexural strength test. The test was carried out with a standard compression testing fixture, as shown in Figure 4. The compressive strength was calculated using Equation (2), and the result was taken as the arithmetic mean of the six valid measurements.

(1) $$\sigma_{f}=\frac{3Fl}{2bh^{2}}$$

where $\sigma_{f}\;$ represents the flexural strength (MPa), *F* denotes the maximum failure load (N), and *l* is the span length (100 mm); *b* and *h* represent the beam sample width and height (both 40 mm).

(2) $$f=\frac{F}{A}$$

where *f* represents the compressive strength (MPa), and *A* is the sample load-bearing area.

#### 2.3.2. Effective Porosity

The effective porosity of the samples was determined in accordance with ASTM C642-13 [22]. The samples were first dried to a constant mass in an oven at 110 ± 5 °C and weighed to obtain the dry mass ($m_{dry}$). They were then immersed in deionized water for 24 h to allow water to fill the open pores. After immersion, the samples were removed, and the surface moisture was gently wiped off with a damp cloth to obtain the saturated surface-dry mass ($m_{sat}$). The volume of open pores ($V_{pores}$) was determined from the mass difference between the saturated surface-dry and dry states, assuming the density of water ($\rho_{w}$) to be 1 g/cm^3^. The effective porosity ($P_{e}$) was calculated using the following equation:

(3) $$P_{e}=\frac{V_{pores}}{V_{b}}\times 100\%=\frac{m_{sat}-m_{dry}}{V_{b}\times \rho_{w}}\times 100\%$$

where $V_{b}$ denotes the sample volume, determined by the water displacement method or dimensional measurement.

For each test group, three replicate specimens were tested, and the mean value was reported.

#### 2.3.3. Volumetric Water Absorption

The volumetric water absorption (*W_v_*), serving as a critical indicator of FCC pore structure, was determined in strict compliance with ISO 12571:2013 [23]. Specimens with dimensions of 100 mm × 100 mm × 100 mm were cut from the central portion of the prismatic samples to ensure uniformity. For each test group, three replicate specimens were tested. The volumetric water absorption was calculated using Equation (4):

(4) $$W_{v}=\frac{m_{g}-m_{b}}{V_{0}\cdot \rho_{w}}\times 100\%$$

where $m_{g}$ represents the mass of water-saturated sample (g), $m_{b}$ is the mass of dry sample (g), $V_{0}$ is the initial sample volume (cm^3^), and $\rho_{w}$ denotes water density (g/cm^3^).

#### 2.3.4. Softening Coefficient

The softening coefficient (*K*) of the FCC was determined in accordance with ISO 12439:2010 standard [24], using 70.7 mm cubic samples. For each test group, a total of six specimens were prepared: Three for dry-state testing and three for saturated-state testing. Three specimens after curing were dried in an oven at 105 ± 5 °C to constant mass (mass change less than 0.1% over 24 h) to obtain the dry specimens. The remaining three specimens were saturated by immersion in deionized water at 20 ± 2 °C for 48 h, as recommended for evaluation of water resistance. The softening coefficient was calculated using Equation (5):

(5) $$K=\frac{f}{F}$$

where *f* represents the compressive strength of the water-saturated sample (MPa), and *F* is compressive strength of the dry sample (MPa).

#### 2.3.5. XRD

To identify the phase composition and hydration products of the cementitious materials, XRD analysis was conducted. Sample preparation involved selecting the central portion of the specimens to ensure representativeness. The selected samples were then manually ground in an agate mortar until a fine powder passing through a 75 μm sieve was obtained. XRD patterns were recorded using a Rigaku Ultima IV X-ray diffractometer (Rigaku Corporation, Tokyo, Japan) with the following acquisition parameters: Cu-Kα radiation (λ = 1.5418 Å), generated at an accelerating voltage of 40 kV and a current of 40 mA. Data were collected over an angular range of 5° to 80° (2θ), with a step size of 0.02° and a scanning speed of 2°/min to ensure adequate resolution. Phase identification was performed by comparing the obtained diffraction patterns with standard patterns from the International Centre for Diffraction Data database. The interpretation focused on identifying the main crystalline phases of the sulfoaluminate cement and monitoring the evolution of its hydration products.

#### 2.3.6. SEM

The microstructural morphology of the specimens was examined using SEM (ZEISS, Oberkochen, Germany). Sample preparation involved taking small fragments from the fractured specimens, each with an area of approximately 1 cm^2^, to expose fresh, unaltered surfaces for observation. SEM imaging was performed using a Zeiss Merlin Compact scanning electron microscope (ZEISS, Oberkochen, Germany). The acquisition parameters were set as follows: An accelerating voltage of 5 kV to balance resolution and sample damage. The interpretation focused on identifying the distribution of hydrophobic materials (e.g., AG, HGM) within the cement matrix, examining the interfacial transition zone between these additives and the hydration products, and evaluating the overall compactness of the microstructure.

## 3. Results and Discussion

### 3.1. Volumetric Water Absorption Rate Test Results

**Figure 5:**
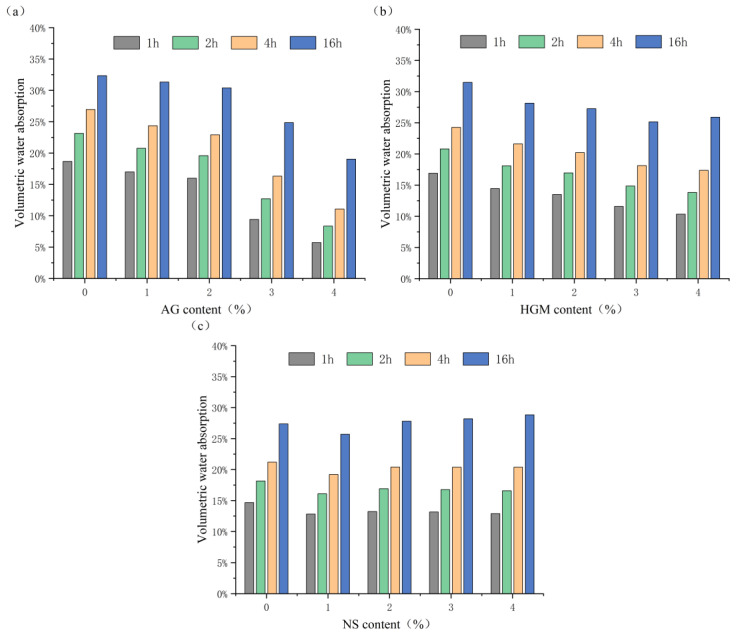
(**a**) AG-induced variation in volumetric water absorption over time; (**b**) HGM-induced variation in volumetric water absorption over time; (**c**) NS-induced variation in volumetric water absorption over time.

The volumetric water absorption of foam concrete is closely related to its pore structure. Moisture ingress is governed by three mechanisms: (1) interconnected pores forming water channels; (2) capillary adsorption in fine pores; and (3) rapid permeation through interfacial defects [25]. Time-dependent absorption curves help evaluate these contributions. Orthogonal experimental results and range analysis were used to assess the influence of AG, HGM, and NS on water absorption over time (Figure 5).

Volumetric water absorption decreased consistently with higher AG content across all intervals (Figure 5a). Maximum reductions reached 69% (1 h), 64% (2 h), and 59% (4 h). Long-term volumetric water absorption (16 h) decreased by up to 41%, respectively, at 4% AG. This is attributed to AG’s hydrophobicity and nano-porosity, which reduce pore connectivity and improve bulk pore structure.

HGM incorporation also reduced volumetric water absorption, with decreases of 39% (1 h), 34% (2 h), and 28% (4 h) (Figure 5b). Long-term volumetric water absorption declined by 20% (16 h) at 3% HGM. These results indicate HGM’s role in modifying both surface and internal pore networks.

As shown in Figure 5c, NS had a limited effect on volumetric water absorption. The maximum impact was observed at a 1% dosage, which reduced the 1-h volumetric water absorption by 13%. However, the effects of NS dosages of 2%, 3%, and 4% were less than 5% and could be considered negligible. This non-linear trend can be attributed to the complex microstructural changes induced by NS. On one hand, the formation of C-S-H gel refines the pore structure, which can reduce pore size. On the other hand, while this refinement may create some discontinuous pores that hinder absorption [26,27], a higher NS content tends to promote the formation of interconnected small pores, ultimately leading to increased volumetric water absorption.

Combined with the variance analysis results in Table 4, the significance value for NS is >0.05, indicating that NS has a negligible effect on the volumetric water absorption by volume of foam concrete. In contrast, the significance values for AG and HGM are both <0.05. Furthermore, the F-value and Type III sum of squares for AG are greater than those for HGM, indicating that AG has a more pronounced influence on the volumetric water absorption by volume of foam concrete than HGM, consistent with the above analysis.

### 3.2. Effective Porosity Test Results

**Figure 6:**
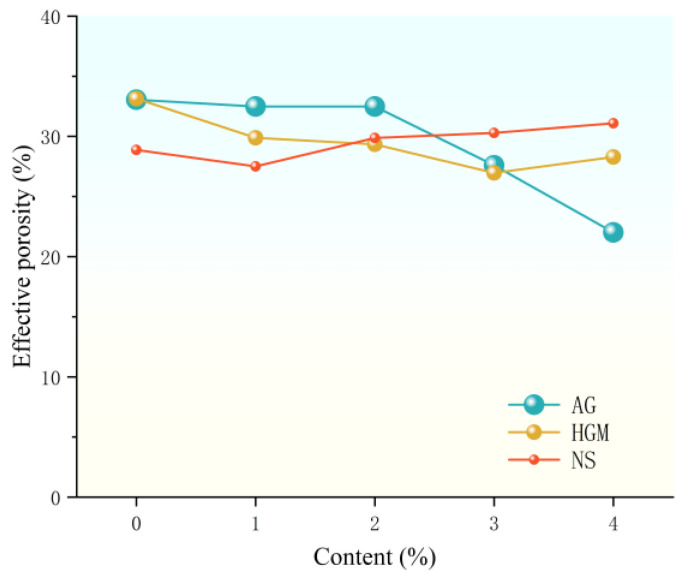
Effects of AG, HGM, and NS content on the effective FCC porosity.

The effective porosity of FCC was evaluated through orthogonal testing with range analysis. As shown in Figure 6, porosity remained stable at low AG content but decreased by up to 33% at higher concentrations. This reduction is attributed to AG’s role in increasing median pore size—where total porosity inversely correlates with pore area [28]—and its defoaming effect due to hydrophobic interfaces [29]. Smaller AG particles enhance defoaming efficiency through greater contact area.

HGM incorporation initially reduced porosity by up to 19%, owing to the replacement of foam with closed, rigid micropores at constant density. Further increases slightly raised porosity.

As the content of NS increases, the effective porosity of foamed concrete initially decreases and then gradually rises, showing an overall upward trend with a maximum increment of 13%. Based on the variance analysis results in Table 4, the significance value for NS is greater than 0.05, with relatively low F-values and mean square, indicating that the effect of NS on the effective porosity of foam concrete can be considered negligible.

**Figure 7:**
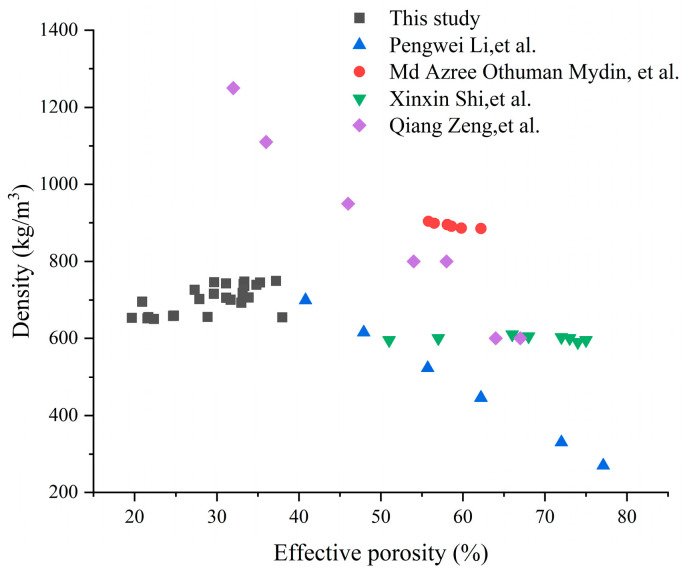
A comparison of the effective porosity recorded in this work with published studies related to similar material density [27,30,31,32].

According to the variance analysis data on effective porosity presented in Table 4, the order of influence on effective porosity was AG > HGM > NS. Compared to literature values at similar densities (Figure 7), our samples showed notably lower porosity, resulting from the use of hydrophobic additives (AG, HGM) and partial replacement of foaming agents, effectively suppressing bubble formation and providing a novel low-porosity FCC design.

### 3.3. Mechanical Properties Test Results

**Figure 8:**
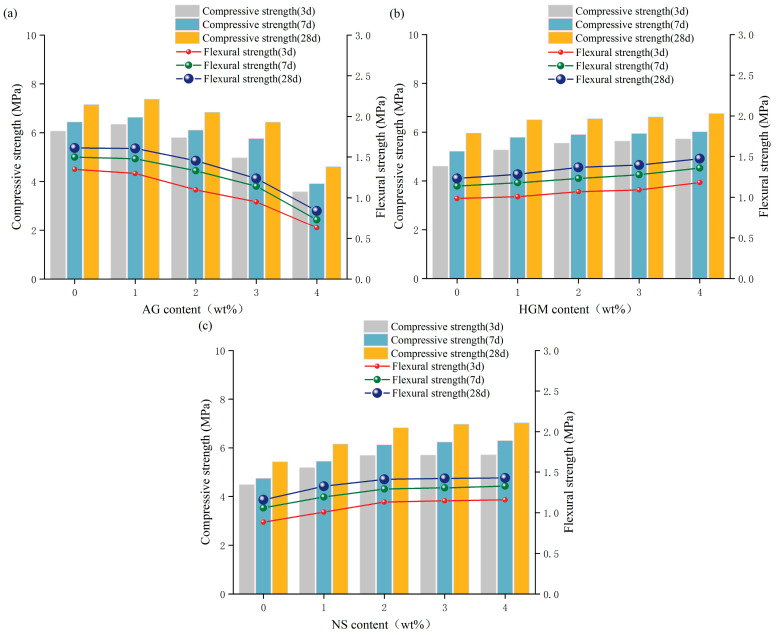
Test results for the effect of material composition on compressive and flexural strength. (**a**) Effect of AG content on compressive and flexural strength; (**b**) Effect of HGM content on compressive and flexural strength; (**c**) Effect of NS content on compressive and flexural strength.

**Figure 9:**
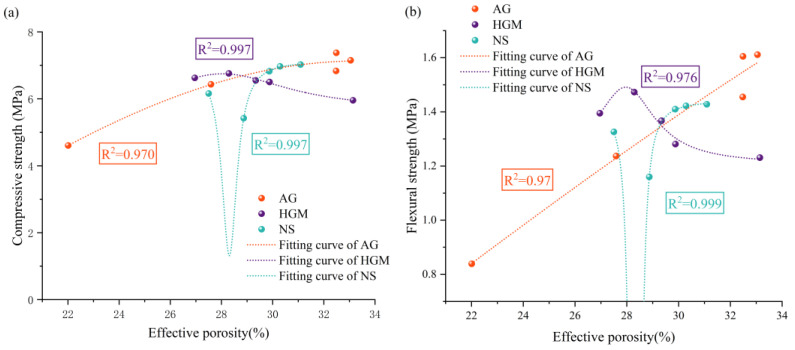
Relationship between effective porosity and mechanical strength. (**a**) Relationship between effective porosity and compressive strength; (**b**) Relationship between effective porosity and flexural strength.

In order to evaluate the influence of AG, HGM, and NS on the mechanical properties of FCC, compressive and flexural strength tests were conducted after 3, 7, and 28 days; the results following range analysis are presented in Figure 8. In addition, the relationship between compressive/flexural strength and effective porosity after 28 days was fitted using Lorentz functions, which serve as empirical tools to describe the observed trend, as shown in Figure 9.

#### 3.3.1. The Influence of AG

The variation in compressive strength of FCC with increasing AG content (Figure 8a) indicates that, as the aging period extends from 3 to 28 days, the compressive strength initially remains largely unchanged before gradually decreasing. A comparison of the 28-day compressive strength of FCC reveals that at an AG content of 1%, the compressive strength increases by 3.1%, a negligible improvement. However, when the AG content is increased to 4%, the compressive strength decreases by 35.6% relative to the group with 0% AG content. In contrast, flexural strength demonstrated a continuous decline with increasing AG content, reaching a maximum reduction of 47.9% at 28 d. The observed mechanical behavior is due to competing mechanisms resulting from the unique material characteristics of AG. The ultra-hydrophobic nano-porous structure reduces cement paste adsorption capacity [33] while enhancing matrix compactness via nano-filling effects [34], accounting for the initial strength increase at moderate dosages. These observations align with the findings of Li et al. [29], who reported an optimal strength at an intermediate AG content. Excessive AG incorporation results in detrimental effects, which include: hydrophobic interference with cement hydration [35], formation of irregular pores, and AG agglomeration creating strength-limiting pore structures [36]. The particularly pronounced decrease in flexural strength reflects a greater sensitivity to pore uniformity and connectivity [37], exacerbated by AG-induced water redistribution effects.

Owing to the intrinsically low mechanical strength and superhydrophobic nature of AG, increasing its content progressively reduces the material’s effective porosity. However, the overall mechanical properties exhibit a declining trend. In general, a reduction in effective porosity is accompanied by an increase in both compressive and flexural strengths. In this case, however, the weak interfacial bonding between AG and cement hydration products, combined with the inherently low strength of AG, leads to a decrease in mechanical strength despite the reduced porosity. As illustrated in Figure 9, the relationships between compressive/flexural strength and effective porosity can be well described by the Lorentz function. The corresponding fitting equations are given in Equation (6) for compressive strength versus effective porosity, and in Equation (7) for flexural strength.

(6) $$y=-21.44+\frac{2\times 32.94}{\pi }\times \frac{0.73}{4\times {\left(x-0.33\right)}^{2}+0.73^{2}}$$

(7) $$y=-5.57+\frac{2\times 17.92}{\pi }\times \frac{1.43}{4\times {\left(x-0.57\right)}^{2}+1.43^{2}}$$

#### 3.3.2. The Influence of HGM

The mechanical properties of FCC improved consistently with higher HGM content, and this enhancement was more pronounced in flexural strength than in compressive strength (Figure 8b). Specifically, compressive strength increased by 9.2% at an HGM content of 1%, while further increases in HGM content yielded only marginal gains. Nevertheless, at a higher dosage of 4%, the 28-day compressive and flexural strengths were improved by 13.5% and 19.7%, respectively, indicating a sustained positive effect. Furthermore, as presented in Figure 9, both compressive and flexural strengths initially increased and then decreased with rising effective porosity, following Lorentzian trends described by Equations (8) and (9).

(8) $$y=5.66+\frac{2\times 0.11}{\pi }\times \frac{0.06}{4\times {\left(x-0.28\right)}^{2}+0.06^{2}}$$

(9) $$y=1.21+\frac{2\times 0.01}{\pi }\times \frac{0.03}{4\times {\left(x-0.28\right)}^{2}+0.03^{2}}$$

As rigid hollow microspheres with inherently high strength, the incorporation of HGM enhances the mechanical properties of FCC through three primary mechanisms: (1) matrix densification and optimized foam distribution via an enhanced bubble wall structure [38]; (2) improved pore structure, achieved through reinforced bubble confinement that reduces bubble diameter and buoyancy [39,40], thereby increasing foam stability and optimizing pore size distribution; (3) increased foam wall thickness and spacing associated with uniform HGM dispersion, which enhances mechanical performance [38]. These findings are consistent with the work of Lu et al. [41] and Lee et al. [42] who successfully applied HGM as a partial foam replacement to produce lightweight high-strength cementitious composites. Therefore, with the incorporation of HGM, the mechanical properties of FCC generally improve with decreasing effective porosity.

#### 3.3.3. The Influence of NS

The addition of NS significantly enhanced the mechanical properties of FCC (Figure 8c), achieving maximum increases of 29.6% in compressive strength and 23.1% in flexural strength. The optimal improvement was achieved at an NS content of 2%, enhancing compressive and flexural strength by 25.9% and 21.6%, respectively, while higher dosages yielded diminishing returns. When plotted as a function of porosity, the mechanical properties exhibited a V-shaped trend (Figure 9), with flexural strength showing greater sensitivity to porosity variations. The relationships were fitted using Lorentz functions, as described by Equations (10) and (11).

(10) $$y=7.13+\frac{2\times \left(-0.07\right)}{\pi }\times \frac{0.01}{4\times {\left(x-0.28\right)}^{2}+0.01^{2}}$$

(11) $$y=1.44+\frac{2\times \left(-0.18\right)}{\pi }\times \frac{2.86}{4\times {\left(x-0.28\right)}^{2}+2.86^{2}}$$

The strengthening effect resulting from NS additions arises from both chemical reactions and physical effects. Chemically, NS participates in pozzolanic reactions with calcium hydroxide to form C-S-H gel [27]. It also acts as nucleation sites, promoting cement hydration and the formation of C-S-H gel with a low calcium-to-silicate ratio [43,44,45]. Physically, the nano-filling effect of NS particles densifies the matrix by reducing pore size and improving pore structure, particularly through the formation of dense C-S-H gel layers on the pore walls. These combined effects account for the observed strength enhancement, for which a 2% NS dosage represents the optimal balance between pore refinement and the onset of particle agglomeration at higher concentrations. The addition of NS effectively modifies both the chemical composition and physical structure of FCC, although these beneficial effects are subject to an upper dosage limit. This underscores the importance of controlled NS incorporation to achieve optimal performance.

Based on the variance analysis results presented in Table 4, the order of influence on mechanical properties is AG > NS > HGM. However, the effect of AG on mechanical performance is adverse, as increasing its content leads to a significant decline in the mechanical properties of foam concrete, whereas the impact of HGM is comparatively minimal.

#### 3.3.4. Comparative Analysis of Published FCC Mechanical Property Data

**Figure 10:**
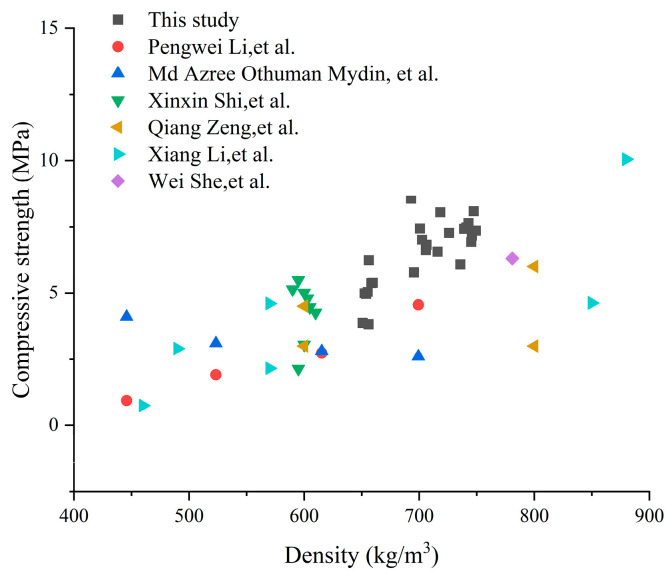
A comparison of the compressive strength recorded in this study with values reported in the literature for related FCC materials with similar density [27,30,31,32,46,47].

**Figure 11:**
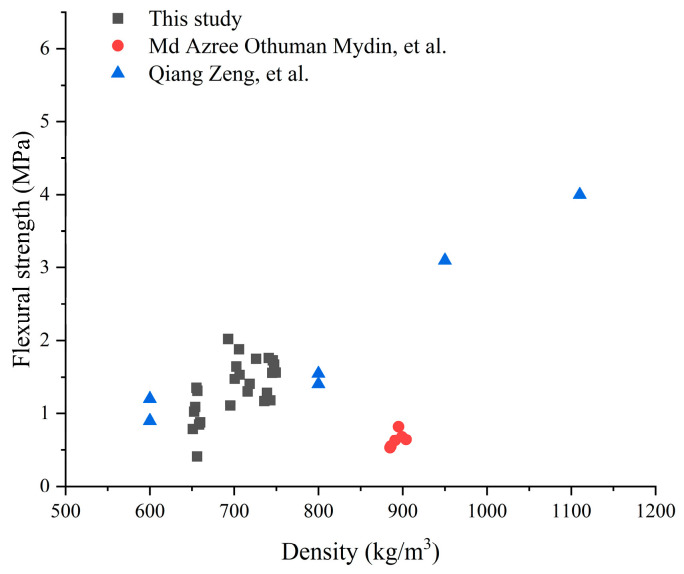
A comparison of the flexural strength recorded in this study with values reported in the literature for related FCC materials with similar density [27,32].

The FCC prepared in this study was designed with a target density of 700 kg/m^3^, and the measured 28-day densities ranged from 650 to 750 kg/m^3^, meeting the standard requirements. Figure 10 and Figure 11 present a comparative analysis of the 25 experimental groups from this study with data reported in the literature for FCC of similar densities.

The 28-day compressive and flexural strengths of most test groups in this study significantly exceed those reported in the recent literature for FCC systems of equivalent density, with only a few groups exhibiting intermediate levels. The observed level of performance can be attributed to three factors: (1) the incorporation of HGM and NS effectively improved mechanical strength; (2) partial replacement of foam with the optimal AG content enhanced compressive strength, while excess AG addition resulted in a significant reduction in strength; (3) AG incorporation consistently lowered flexural strength, an effect that contrasts with its influence on compressive strength.

### 3.4. Softening Coefficient Test Results

**Figure 12:**
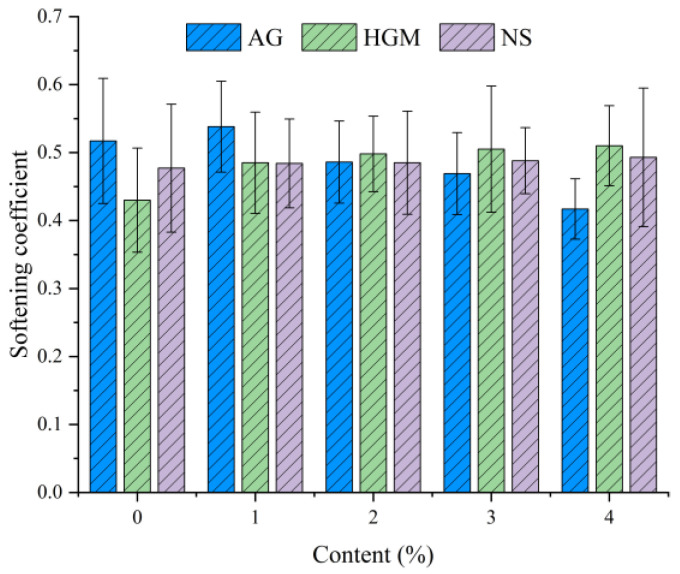
The softening coefficient of FCC as a function of material composition.

The softening coefficient, which reflects the strength retention of FCC under water saturation, was analyzed using orthogonal range analysis. The results are presented as the mean of range analysis ± standard deviation in Figure 12, based on triplicate specimens. The corresponding analysis of variance is shown in Table 4 to assess the statistical significance of the observed differences.

With 1% AG addition, the coefficient increased marginally by 4.1% compared to the control. However, analysis of variance indicated that this effect, as well as the subsequent reduction of up to 19.3% at higher AG contents, was not statistically significant (*p* = 0.138 > 0.05). This suggests that the variations induced by AG fall within the range of normal measurement variability. This may be attributed to the dual role of AG: at low dosages, its hydrophobic and nano-porous characteristics can fill capillary pores and inhibit water adsorption. At higher dosages, however, excessive AG may create interconnected capillary networks, which induce pore pressure under loading and weaken interfacial bonding [36]. The net effect is insufficient to produce a statistically significant change in the softening coefficient under the current experimental conditions.

HGM consistently improved the softening coefficient, with a maximum enhancement of 18.6% relative to the control. Despite this apparent improvement, the analysis of variance results (*p* = 0.44) indicates that the differences are not statistically significant due to the variability among replicate measurements, as reflected by the error bars in Figure 12. Although the sealed hollow structure of HGM reduces permeable voids, its uniform dispersion refines the pore structure, and its chemical inertness enhances interfacial stability under saturated conditions, the experimental scatter suggests that the magnitude of improvement is not robust enough to achieve statistical significance.

NS showed a negligible influence, with a maximum increase of only 3.4%. This finding aligns with the analysis of variance results (*p* = 0.998), confirming no significant effect. It is also consistent with its limited effect on water absorption (Figure 5c), as NS neither significantly alters transport pathways nor disrupts the saturated strength of FCC.

In summary, although the addition of AG and HGM led to certain improvements in the softening coefficient of FCC, these changes were not statistically significant based on analysis of variance results (*p* > 0.05), indicating that the observed variations may be attributed to normal experimental variability rather than material effects. NS exhibited no discernible influence, consistently aligning with its negligible role in altering water transport or saturated strength.

### 3.5. Microstructure Analyses

#### 3.5.1. Morphological Observation

**Figure 13:**
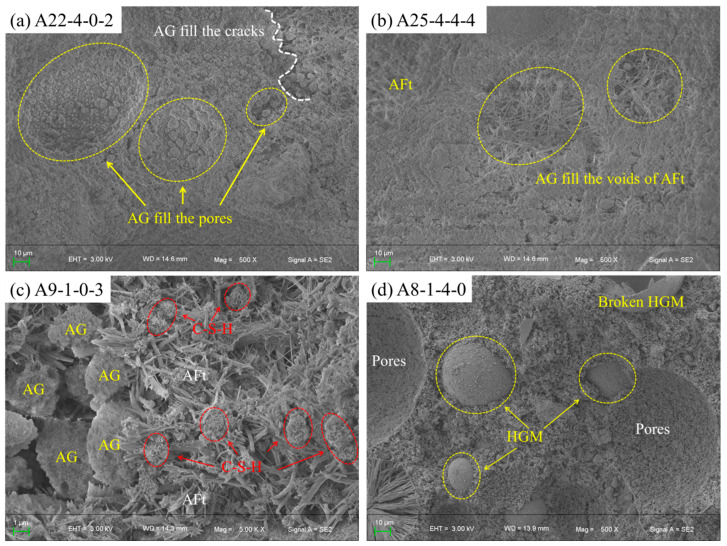
Representative SEM images of FCC. (**a**) SEM image of specimen A22-4-0-2; (**b**) SEM image of specimen A25-4-4-4; (**c**) SEM image of specimen A9-1-0-3; (**d**) SEM image of specimen A8-1-4-0.

After compressive strength testing, selected specimens A8-1-4-0, A22-4-0-2, and A25-4-4-4 were examined at 500× magnification, while A9-1-0-3 was observed at 5000× for microstructural analysis (Figure 13).

As shown in Figure 13a, AG particles effectively filled micrometer-scale pores and microcracks within the cement hydration products. Due to the use of rapid-hardening sulfoaluminate cement, AG was uniformly dispersed among hydration products such as AFt during mechanical stirring (Figure 13b). However, with increasing hydrophobic AG content, the interfacial bonding deteriorated, resulting in a distinct weak interfacial transition zone and AG agglomeration (Figure 13c).

In contrast, Figure 13d shows that HGM exhibited good interfacial compatibility with the cement matrix. In crushed specimens, HGM particles typically fractured rather than debonded, indicating strong interfacial adhesion.

Comparison of Figure 13a,b,d at the same magnification indicates that the compactness of the cement matrix follows the order: Figure 13b > a > d. The density difference between mixes with 2% NS (Figure 13b) and 4% NS (Figure 13d) is minor, which is consistent with the mechanical results in Figure 8c, indicating that increasing NS from 2% to 4% did not substantially enhance properties. This is because NS significantly improves matrix density even at low contents. Previous studies have confirmed that optimal mechanical performance in foam concrete is achieved with 2% NS [48,49] or 3% NS [27,50]. The high reactivity of NS arises from its silanol-rich surface (Si–OH), which participates in pozzolanic reactions, consumes Ca(OH)_2_, and forms additional chain-structured C–S–H gel [51]. These chains interweave into a network, increasing structural density [52]. The nano-structured C–S–H gel exhibits higher polymerization, enhancing the mechanical properties and durability of cement-based materials [51].

#### 3.5.2. Mineralogical Characteristics

**Figure 14:**
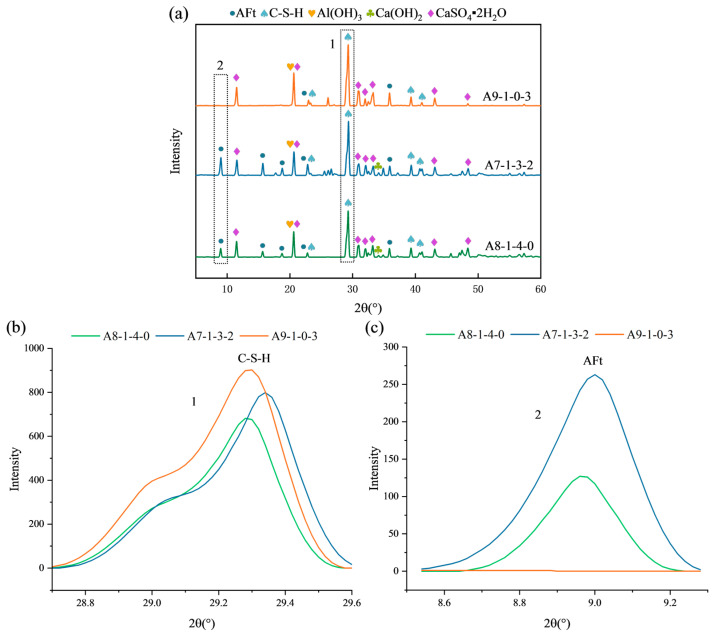
Effect of NS and HGM addition on the XRD patterns (A7–A9). (**a**) XRD patterns of specimens A7–A9; (**b**) Enlarged view of C-S-H patterns; (**c**) Enlarged view of AFt patterns. Note: (**b**,**c**) are the magnified patterns of regions 1 and 2 in (**a**), respectively.

Following 28-day curing and compressive strength tests, selected specimens A7-1-3-2, A8-1-4-0, and A9-1-0-3 were ground for XRD analysis (Figure 14). The main phases identified include calcium silicate hydrate (C–S–H, PDF#99-000-1915), ettringite (AFt, PDF#99-000-1074), aluminum gel (Al(OH)_3_, PDF#99-000-1340), Ca(OH)_2_ (PDF#99-000-2999), and CaSO_4_·2H_2_O (PDF#04-009-3817). To evaluate the evolution of the crystalline phases (AFt, CH, etc.), the peak intensity ratios of their major diffraction peaks relative to the strongest peak of each specimen were analyzed. It is important to note that C–S–H is primarily a poorly crystalline or amorphous phase [53], which typically exhibits broad humps rather than sharp diffraction peaks in XRD patterns. Therefore, while the characteristic hump of C–S–H around 2θ = 29.3° can indicate its presence, any comparison of its “intensity” should be interpreted qualitatively or semi-quantitatively at best, as it does not represent a well-defined crystal structure.

As shown in Figure 14a, the characteristic C–S–H hump becomes more pronounced with higher NS content, suggesting an increased formation of C–S–H gel. This qualitative observation is consistent with the strength development discussed earlier. In contrast, the crystalline phases exhibit distinct trends. Although the C–S–H peak of HGM-containing specimens (A7-1-3-2 and A8-1-4-0) is less pronounced than that of A9-1-0-3 (without HGM), Figure 14a shows that the number and intensity of AFt peaks in HGM-containing specimens are significantly higher. The highest AFt diffraction peaks were selected for comparison (Figure 14c). The peak intensity ratio of the main AFt peak (2θ = 9°) increased from 0 in A9-1-0-3 to 127 in A8-1-4-0 and 263 in A7-1-3-2. This confirms that HGM addition significantly promotes the formation of crystalline AFt. This phenomenon can be attributed to the reactive silica-based shell of HGM [54], which may provide additional Al and Si species to facilitate AFt formation.

## 4. Further Discussion

### 4.1. Orthogonal Experiment

Based on the range and variance analysis of the orthogonal experiments, the following conclusions can be drawn: For water absorption by volume and effective porosity, the order of influence is AG > HGM > NS, and all three factors reduce these properties. For mechanical performance, the order of influence is AG > NS > HGM, with AG having a detrimental effect, while NS and HGM enhance it. For the softening coefficient, the order of influence is AG > HGM > NS, with AG reducing the softening coefficient, while HGM and NS improve it.

As reported in the literature [12], under the same mix proportion, materials, and testing methods, the order of influence on thermal conductivity is AG > HGM > NS. Specifically, AG and HGM reduce thermal conductivity: 4% AG decreases thermal conductivity by 44.29%, and 4% HGM decreases it by 17.13%. In contrast, 1% NS increases thermal conductivity by 12.54%. However, when the NS content increases from 2% to 4%, the thermal conductivity increases only marginally by 3.41%.

As noted earlier, excessive incorporation of NS (3–4%) has a limited effect on improving the mechanical performance of foam concrete. Therefore, based on the above considerations, the optimal mix ratio for AG, HGM, and NS was determined to be 4% AG + 4% HGM + 2% NS (designated as A4H4N2).

Using this proportion, foam concrete with a target dry density of 700 kg/m^3^ was prepared. The performance results (Table 5) show that the A4H4N2 mixture exhibits superior effective porosity, compressive strength, flexural strength, and softening coefficient compared to samples containing only 4% AG (e.g., A21-4-1-0, A22-4-0-2, A23-4-2-3, A24-4-3-1, and A25-4-4-4). Moreover, its thermal conductivity, measured via the transient plane source method (TC5100, Xi’an Xiaxi Electronic Technology Co., Ltd., Xi’an, China), is 0.0824 W/m·K (as shown in Table 5). For context, this value is lower than that of mixtures incorporating only 4% AG, 4% HGM, or 4% NS (e.g., mixture A25-4-4-4), which exhibited a thermal conductivity of 0.1071 W/m·K under the same testing conditions in our previous study [12].

### 4.2. Synergistic Effects of AG, HGM, and NS on FCC

Hydrophobic AG exerts a dual influence on the mechanical properties of FCC. At lower dosages, its nano-scale particles fill pores and reduce effective porosity, mitigating the adverse effects of its low intrinsic strength and weak interfacial bonding, thereby maintaining or even slightly improving compressive and flexural strength. However, with increased AG content, these negative effects dominate. Although pores within AG agglomerates do not form continuous channels, the degradation of mechanical properties—the promotion of AFt formation experimental results and references [55,56]—is primarily due to poor adhesion to the cement matrix and the low strength of AG. Thus, despite its significant thermal insulation benefits, AG dosage should be limited for both performance and cost reasons. Thermal performance can be optimized by partially replacing AG with cost-effective HGM.

**Figure 15:**
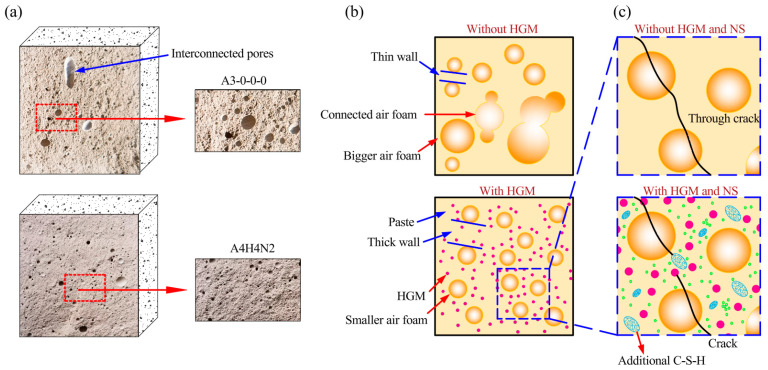
Schematic representation of the FCC mechanical strength enhancement due to HGM and NS addition. (**a**) Fracture surfaces of specimens A3-0-0-0 and A4H4N2; (**b**) Schematic illustration of the FCC structure with and without HGM; (**c**) Schematic illustration of FCC structure with and without HGM and NS during crack initiation.

HGM contributes to the mechanical properties of foam concrete through improved interfacial bonding, optimized pore structure, and the promotion of AFt formation. A comparison of the fracture surfaces of plain foam concrete (A3-0-0-0) and the optimal mixture (A4H4N2) is shown in Figure 15a. Based on SEM observations, the incorporation of HGM was observed to refine foam size, increase pore spacing, and improve pore size distribution [38] (Figure 15b). These microstructural changes are believed to reduce interconnected porosity, increase wall thickness, and alleviate stress concentration, thereby contributing to enhanced mechanical performance.

NS is also believed to improve mechanical properties by increasing matrix compactness. Due to its high specific surface area and pozzolanic activity, NS can act as nucleation sites and react with Ca(OH)_2_ to form additional C–S–H gel, which refines the microstructure [51].

The proposed multi-scale synergistic enhancement mechanism is schematically illustrated in Figure 15c. Based on qualitative microstructural observations, in the absence of HGM and NS, continuous cracks tend to form around pores, which may impair mechanical performance. In contrast, HGM is observed to hinder crack propagation through its high-strength hollow structure, whereas NS contributes through the formation of secondary C–S–H. This synergy is proposed to delay crack formation and enhance the mechanical properties of foam concrete.

## 5. Conclusions

Orthogonal experiments were conducted to evaluate the effects of AG, HGM, and NS on the properties of FCC. The key findings are summarized as follows:

(1) AG reduced the volume water absorption, effective porosity, softening coefficient, and mechanical properties, with the deterioration in mechanical performance becoming more pronounced at dosages exceeding 1%.
(2) HGM decreased both volume water absorption and effective porosity (with the latter reduced by up to 19%), while simultaneously enhancing mechanical properties with increasing dosage. Compressive and flexural strengths increased by up to 13.5% and 19.7%, respectively, and the softening coefficient showed an apparent improvement of 18.6%.
(3) NS significantly enhanced mechanical performance, increasing compressive and flexural strengths by 29.6% and 23.1%, respectively, but exhibited no notable influence on water absorption or the softening coefficient, while marginally increasing porosity.
(4) Based on the above performance characteristics and considering the effects of AG, HGM, and NS on thermal insulation, the optimal mixture was determined as 4% AG, 4% HGM, and 2% NS (designated A4H4N2). This formulation achieves a balance between thermal insulation and mechanical properties, satisfying the requirements for exterior wall insulation materials.

## Figures and Tables

**Table 1:** Structural and performance parameters for sulphoaluminate cement.

Performance Index	Specific Surface Area	pH	Free Expansion Rate	Initial Setting Time	Final Setting Time
Sulphoaluminate cement	≥400 m^2^/kg	10.5 (after water addition, 1 h)	0.00%~0.10%	6 min	11 min

**Table 2:** Structural and performance parameters for hydroxypropyl methylcellulose.

Admixture	Bulk Density (g/cm^3^)	Density (g/cm^3^)	Viscosity (MPa·s)	Hydroxypropyl Value(%)	Methoxyl Value (%)
HPMC	0.4	1.26	200,000	11.5	26

**Table 3:** Orthogonal experiment scheme and results.

Label	Factor(m/m%)			Foam Volume Fraction	Compressive Strength (MPa)			Flexural Strength (MPa)		
	AG	HGM	NS		3 d	7 d	28 d	3 d	7 d	28 d
A1-0-2-1	0%	2%	1%	39.7%	5.74	6.15	6.81	1.11	1.32	1.53
A2-0-4-2	0%	4%	2%	31.6%	7.57	7.76	8.57	1.75	1.86	2.02
A3-0-0-0	0%	0%	0%	48.3%	3.69	4.25	5.04	1.05	1.24	1.35
A4-0-1-3	0%	1%	3%	37.6%	6.96	7.34	8.05	1.26	1.37	1.404
A5-0-3-4	0%	3%	4%	29.6%	6.35	6.70	7.28	1.58	1.7	1.75
A6-1-1-1	1%	1%	1%	39.0%	5.96	6.26	7.16	1.22	1.51	1.73
A7-1-3-2	1%	3%	2%	30.9%	6.77	6.96	7.64	1.01	1.15	1.18
A8-1-4-0	1%	4%	0%	32.9%	5.61	5.89	6.62	1.53	1.76	1.88
A9-1-0-3	1%	0%	3%	36.9%	6.27	6.69	7.36	1.35	1.41	1.564
A10-1-2-4	1%	2%	4%	29.0%	7.09	7.36	8.09	1.38	1.56	1.67
A11-2-1-4	2%	1%	4%	28.3%	5.60	6.04	6.93	1.27	1.47	1.56
A12-2-0-1	2%	0%	1%	38.3%	5.23	5.54	6.08	1	1.12	1.17
A13-2-2-2	2%	2%	2%	30.2%	6.63	6.87	7.48	1.38	1.58	1.76
A14-2-4-3	2%	4%	3%	22.6%	6.19	6.65	7.44	1.04	1.38	1.474
A15-2-3-0	2%	3%	0%	32.2%	5.30	5.45	6.24	0.8	1.11	1.31
A16-3-0-4	3%	0%	4%	27.6%	5.07	6.56	7.43	0.92	1.2	1.28
A17-3-2-0	3%	2%	0%	31.5%	4.73	4.89	5.38	0.68	0.8	0.85
A18-3-3-3	3%	3%	3%	22.0%	5.52	6.32	7.01	1.29	1.48	1.644
A19-3-4-1	3%	4%	1%	23.8%	4.78	4.97	5.78	0.94	1.08	1.11
A20-3-1-2	3%	1%	2%	29.5%	4.72	6.04	6.56	0.92	1.15	1.3
A21-4-1-0	4%	1%	0%	30.8%	3.08	3.22	3.82	0.36	0.39	0.41
A22-4-0-2	4%	0%	2%	28.8%	2.72	3.01	3.87	0.6	0.72	0.79
A23-4-2-3	4%	2%	3%	21.3%	3.51	4.21	4.99	0.79	0.89	1.024
A24-4-3-1	4%	3%	1%	23.1%	4.17	4.28	4.96	0.77	0.94	1.09
A25-4-4-4	4%	4%	4%	14.1%	4.41	4.83	5.39	0.64	0.71	0.88

Note: All compressive strength and flexural strength values presented are mean results. For each group, *n* = 3 for compressive strength and *n* = 6 for flexural strength.

**Table 4:** Analysis of variance results.

Source of Variance	Dependent Variable	Type III Sum of Squares	Degrees of Freedom	Mean Square	F-Value	Significance
AG	16 h volumetric water absorption	0.063	4	0.016	20.009	<0.001
	Effective porosity	0.045	4	0.011	14.525	<0.001
	28 d compressive strength	24.438	4	6.11	18.598	<0.001
	28 d flexural strength	2.006	4	0.523	6.453	0.005
	Softening coefficient	0.044	4	0.011	2.143	0.138
HGM	16 h volumetric water absorption	0.012	4	0.003	3.895	0.03
	Effective porosity	0.011	4	0.003	3.44	0.043
	28 d compressive strength	1.899	4	0.475	1.445	0.279
	28 d flexural strength	0.147	4	0.045	0.561	0.695
	Softening coefficient	0.021	4	0.005	1.011	0.44
NS	16 h volumetric water absorption	0.003	4	0.001	0.89	0.499
	Effective porosity	0.004	4	0.001	1.24	0.346
	28 d compressive strength	9.408	4	2.352	7.16	0.003
	28 d flexural strength	0.246	4	0.064	0.796	0.55
	Softening coefficient	0.001	4	0	0.031	0.998

**Table 5:** Test results of performance of specimens with the optimal mix proportion.

Label	Dry Density	Effective Porosity	Compressive Strength (28 d)	Flexural Strength (28 d)	Softening Coefficient	Thermal Conductivity
A4H4N2	672 kg/m^3^	26.73%	4.62 MPa	1.03 MPa	0.465	0.0824 W/m⋅K

## Data Availability

The original contributions presented in this study are included in the article. Further inquiries can be directed to the corresponding author.
